# Efficacy of different doses of ketamine as a bolus in major depressive disorder

**DOI:** 10.22088/cjim.9.3.220

**Published:** 2018

**Authors:** Farzan Kheirkhah, Gooya Tayyebi, Seyed Mozaffar Rabiee, Ali-Akbar Moghadamnia, Ali Bijani

**Affiliations:** 1 Social Determinants of Health Research Center, Health Research Institute, Babol University of Medical Sciences, Babol, Iran; 2 Student Research Committee, Babol University of Medical Sciences, Babol, Iran; 3 Cancer Research Center, Health Research Institute, Babol University of Medical Sciences, Babol, Iran; 4 Cellular and Molecular Biology Research Center, Health Research Institute, Babol University of Medical Sciences, Babol, Iran; 5 Non-Communicable Pediatric Diseases Research Center, Health Research Institute, Babol University of Medical Sciences, Babol, Iran

**Keywords:** Ketamine, Major depressive disorder, Bolus, Infusion

## Abstract

**Background:**

Major depressive disorder is a severe, heterogeneous, common medical illness and a leading cause of disability throughout the world that poses a significant public health issue. Previous studies have shown rapid antidepressant effects following a single administration of ketamine. This study aimed to assess the impact of route of administration and dose of ketamine for the reduction of depressive symptoms and compare the effects of different doses and methods.

**Methods:**

A double-blind clinical controlled trial was done on 100 patients with a primary diagnosis of major depressive disorder who were assigned into two groups of 50 subjects at a dose of 0.5 mg/kg and 0.75 mg/kg ketamine and each group was divided into two groups of 25 subjects following a single dose of intravenous bolus and infusion of ketamine. The patient’s severity of depression was evaluated with Hamillton Depression Rating Scale and Beck Depression Inventory scores after 2 days, 7 days, 30 days and 60 days of ketamine administration, then the results were compared between groups.

**Results:**

According to Hamilton and Beck score, the treatment response in investigated patients was 64% and 60%, respectively.

**Conclusions:**

These data suggest that ketamine effect is related to drug dose and type of administration. The dose of 0.75 mg/kg of ketamine is more effective than 0.5 mg/kg and a bolus injection of low-dose ketamine (0.5 mg/kg) is more effective than infusion and in high-dose ketamine (0.75 mg/kg), there was no difference between the methods of drug administration.

Major depressive disorder (MDD) is a severe, heterogeneous, common medical illness and a leading cause of disability throughout the world (1). With its high prevalence, disability, morbidity and mortality, depression poses a significant public health issue (2-4). Recently, the glutamate system has emerged as a critical focus of novel therapeutic development for depressive disorder and particularly treatment-resistant depression (5, 6). Yet, only half of individuals with major depressive episodes respond to the traditional antidepressants, that full clinical benefit of these is only achieved following weeks to months of treatment (7, 8). Therefore, with the challenges of existing pharmacotherapies, there is a clear and urgent need for rapid acting antidepressants with robust efficacy in patients especially who are refractory to traditional antidepressants (9). Ketamine, a noncompetitive N-methyl-D-aspartate (NMDA)-receptor antagonist and FDA-approved anesthetic, is the prototype for a new generation of glutamate-based antidepressants that rapidly alleviate depression within hours of treatment (1). These rapid and potent antidepressant effects were also demonstrated in patient groups known to respond poorly to current antidepressants (10, 11). 

Researchers found that low (subanesthetic) doses of this drug administered intravenously began to reduce depression symptoms within 4 h of administration in patients with severe treatment-resistant depression (12). This finding has since been replicated in multiple-controlled studies by several research groups (13, 14). Studies reported rapid antidepressant effects following a single administration of various routes and doses of ketamine, including 0.2 mg/kg intravenous bolus (15), 50 mg intranasal (16), 0.5 mg/kg or 0.25 mg/kg intramuscular injection (17) and 0.5 mg/kg of ketamine intravenously infused over 40 min (18). NMDA receptor functions vary according to their subunit composition and subcellular location. Convergent evidence shows opposing effects of synaptic and extra synaptic NMDA receptors. Synaptic NMDA receptors promote synaptic formation and neuronal survival. In contrast, extra synaptic NMDA receptor activation promotes synaptic atrophy and neuronal death by altering nuclear calcium. This deregulates target gene expression, leading to mitochondrial dysfunction, reduced dendritic length and arborization, and synaptic loss (1). 

The actions of ketamine are unique. Ketamine is the first step in a cascade of events that includes rapid increases in presynaptic glutamate release, enhanced regional activity in excitatory networks, and ultimately marked changes in synaptic plasticity and connectivity (19-21). Given the known role of BDNF in the pathophysiology and treatment of depression, large trial did find a positive relationship between ketamine effects and peripheral BDNF levels and a polymorphism of the BDNF gene (1). Four hours post ketamine infusion, a significant increase in plasma BDNF was observed in responders compared to non-responders (22-25). Recent studies have concluded that single dosage regimen of intravenous, oral, intranasal and intramuscular ketamine were useful for treating unipolar and bipolar depression (16, 17). Several recent meta-analyses have also shown ketamine to be effective in managing depressive symptoms (13, 14, 26-28). 

We designed the present study to test the rapid antidepressant efficacy of ketamine in a relatively large group of subjects with treatment-resistant major depression. This study aimed to assess the impact of route of administration and dose of ketamine for the reduction of depressive symptoms and compares the effects of different doses and methods. We hypothesized that 0.75 mg/kg ketamine and bolus method would be superior to 0.5 mg/kg ketamine and infusion method in improving depressive symptoms 24 hours following a single injection.

## Methods

The study enrolled patients at one academic site in Babol, Iran, (Yahyanejad Hospital), between January 2014 and September 2015. Patients were eligible to participate if they were 20 to 60 years of age, had a primary diagnosis of major depressive disorder as assessed with the Structured Clinical Interview for DSM-5 patient edition (29).

Additional study inclusion criteria included a history of at least one major depressive episode according to DSM-5 criteria with different severity and without psychotic feature and over the last month are not under medical treatment. Patients were excluded if they had a lifetime history of a psychotic illness, bipolar disorder, somatoform disorder or personality disorder, alcohol or substance abuse in the previous 2 years, medical illness such as hypertension and heart disease, serious and imminent suicidal behavior or homicidal risk, or a score less than 27 on the Mini-Mental State Examination (29) or if they were taking contraindicated medications. Each patient had a physical examination, routine hematologic and biochemical tests, urine toxicology measurements, and an electrocardiogram (ECG) to detect unstable medical illness or substance use. After complete description of the study to the subjects, written informed consent was obtained.

The study patients were free of concomitant antidepressants and other psychotropic medication for the duration of the study and were drug-free prior to the injection, for at least 4 weeks. In this study, 100 patients with major depressive disorder included 50 women with an average age of years 45.82±10.02 (50%) and 50 men with an average age of 35.38±10.19 years (50%), every other one was randomly selected and assigned into two groups of 50 subjects at a ketamine hydrocholoride (ROTEXMEDCA, GERMANY) dose of 0.5mg /kg and 0.75mg/kg and each group was divided into two groups of 25 subjects following a single dose of intravenous bolus and infusion of ketamine infused over 20 minutes. 

Following admission to a clinical research unit and an indwelling catheter was placed in the antecubital vein of the non-dominant arm, and pulse, blood pressure and ECG monitoring were instituted. Patients were discharged from the research unit 4 hours after the injection and were clinically interviewed by a clinical psychologist, 48 hours, 7 days, 30 days and 60 days post injection. Non-responders were considered patients with less than 50% improvement from baseline in the score on the Hamillton Depression Rating Scale (HDRS) (30) and Beck Depression Inventory scores (BDI) (31). As directed by the protocol, we stopped following the nonresponders 48 hours after the injection. Responders were followed until relapse or for an additional 60 days, whichever came sooner.

Data using statistical software SPSS 17 and a test of the model X 2, ANOVA, Fishers and logistic regression were analyzed. Crude and adjusted odd ratios were presented at 95% confidence level. All statistical tests used a threshold of p≤0.05 for significance.

The method for determining the sample size:

$n=\frac{{\left(\mathrm{Z1}-\frac{\alpha }{2}+\mathrm{Z1}-\beta \right)}^{2}(\mathrm{p1q1}+\mathrm{p2q2})}{{\left(\mathrm{p1}-\mathrm{p2}\right)}^{2}}$ =25

(Z_1-__α__/2 _) =1.96

(Z _1-__β_)=0.84 

P_1_ = Improvement in 0.75mg/kg group 

P_2_ = Improvement in 0.5mg/kg group

Sample size was estimated at 95% confidence level and 80% strength, with an assumption of recovery in group 0.75 mg 85% and recovery in 0.5 mg group with 50% sample size for each group of 25 specimens.

This study was registered by Iranian Registry of Clinical Trials (IRCT2015030921072N2). The study was approved by the Ethics Committee of Babol University of Medical Sciences on the nineteenth of March, 2015 (license number 5262).

## Results

Patients were eligible to participate if they were 20 to 60 years of age (average age of 40.6±11.34 years), had a primary diagnosis of major depressive disorder.

Since the response to treatment with ketamine reduced 50% or more of the Hamilton and Beck test scores in patients considered based on the results of the test scores observed in all patients Hamilton noted that based on testing, 64% and 60% of patients respond to treatment, Beck said. Also, fifty patients who underwent injections of 0.5 mg/kg of ketamine were given the Hamilton test results in 24 (48%) patients and according to the Beck test results, 22 (44%) responded to treatment. Fifty patients who underwent injection of 0.75 mg / kg of ketamine were given the Hamilton test results of 40 (80%) patients and while 38 (76%) patients according to the Beck test results. So, a dose of 0.75 mg/kg is more effective than 0.5 mg/kg. In a dose of 0.75 mg/kg, there is no difference between the route of injection and at a dose of 0.5 mg/kg, bolus method is effective respond to treatment (tables 1, 2).

**Figure 1:**
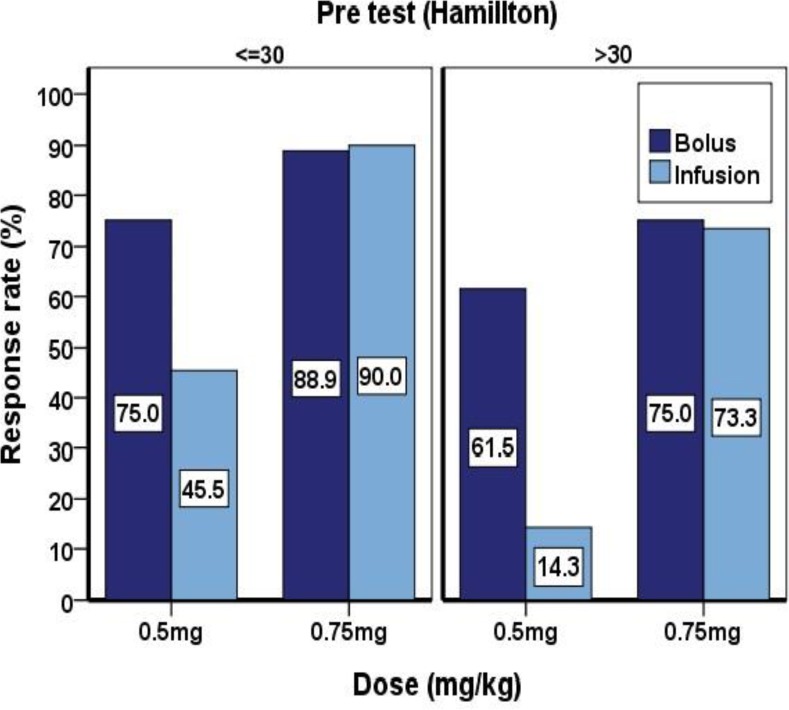
Comparison of the effects of ketamine with respect to dose and injection method in depression varying intensities

In this study, fifty-three patients aged forty years or less and forty-seven patients more than forty years old and 35 patients of the 53 patients and 29 patients out of 47 patients responded to treatment, according to Hamilton test. Of the 64 patients, 34 (53.1% were responding to treatment) females and 30 (46.9% were responding to treatment) males responded to treatment and no significant relationship was observed between age, gender, and response to treatment (p<0.05). In this study, Hamilton patients test scores had 30 as severe major depressive disorder and from the 100 patients, 58 subjects in this group had a 51.6% response to treatment, while the remaining 48.4% of 42 patients had responded to treatment, therefore, patients with severe major depression have lower response to ketamine (fig 1).

Ketamine positive response to the first two days was 64% and 63% of the first week (one relapse) and 59% (five relapses) of the first month respectively. 25 patients did not return for follow-up in the second month and of the 39 patients, 33 patients still retained a positive response.

**Table 1:** Baseline characteristics of studied population

	**Bolus 0.5mg** **n=25**	**Infusion 0.5mg** **n=25**	**Bolus 0.75mg** **n= 25**	**Infusion 0.75mg** **n= 25**	**P value**
Sex					
Male (%) Female (%)	15 (60%) 10 (40%)	11 (44%) 14 (56%)	14 (56%) 11 (44%)	10 (40%) 15 (60%)	0.437
Age (mean ± SD)	40.84±11.75	39 ±11.49	42.84±12.17	39.72±10.18	0.658
Baseline Hamilton test scores (mean ± SD)	32.64±8.27	33.16±9.27	35.32±10.04	33.52±7.6	0.730
Baseline Beck test scores (mean ± SD)	34.84±10.56	33.76±11.87	36.04±11.56	34.44±9.42	0.901

**Table 2:** Prevalence and frequency of treatment response based on Beck and Hamilton, according to the dosage and injection of ketamine

**Group**		**R.H** ¹		**Total**	**R.B** ¹		**Total**
		² **R(+)**	² **R(-)**		² **R(+)**	² **R(-)**	
Bolus 0.5mg %within Group %within Response	Count	17 68% 26.6%	8 32% 22.2%	25 100% 25%	15 60% 25%	10 40% 25%	25 100% 25%
Infusion 0.5mg %within Group %within Response	Count	7 28% 10.9%	18 72% 50%	25 100% 25%	7 28% 11.7%	18 72% 45%	25 100% 25%
Bolus 0.75mg %within Group %within Response	Count	20 80% 31%	5 20% 13.9%	25 100% 25%	19 76% 31.7%	6 24% 15%	25 100% 25%
Infusion 0.75mg %within Group %within Response	Count	20 80% 31%	5 20% 13.9%	25 100% 25%	19 76% 31.7%	6 24% 15%	25 100% 25%
Total %within Group %within Response	Count	64 64% 100%	36 36% 100%	100 100% 100%	60 60% 100%	40 40% 100%	100 100% 100%

¹: reduction of fifty percent and more in the Beck test scores (R.B) and Hamilton test scores (R.H)

² : Positive Response (+), negative Response (-)

## Discussion

These data suggest that ketamine effect is related to drug dose and type of administration. The dose of 0.75 mg/kg of ketamine is more effective than 0.5 mg/kg and a bolus injection of low-dose ketamine (0.5 mg/kg) is more effective than infusion and in high-dose ketamine (0.75 mg/kg), there was no difference between methods of drug administration. We were aware of prior evidence implicating N-methyl-D-aspartate (NMDA) receptors in the pathophysiology and treatment of depression (32, 33), ketamine is a noncompetitive N-methyl-D-aspartate (NMDA) glutamate receptor antagonist. The antidepressant effects emerged so rapidly following the administration of a single ketamine dose and persisted for so long (12). The antidepressant effects tend to emerge 1–2 h after the acute perceptual disturbances of ketamine have abated and can persist for two weeks or longer in some patients (34). In this study, the antidepressant effects of ketamine persisted 64% for two days, 63% for one week and 59% for one month. It is not clear what percentage of the NMDA receptor for antidepressant effects of ketamine dose should be taken and what dose of ketamine can achieve it (35). More specifically, a series of recent studies in rodents has demonstrated that low-dose ketamine administration rapidly triggers three consecutive events: first, a presynaptic disinhibition of glutamatergic neurons, which leads to a glutamate surge; second, an increased activation of the AMPA glutamate receptor, combined with the blockade of extrasynaptic NMDA receptors; and third, a postsynaptic activation of neuroplasticity-related signaling pathways involving BDNF and mTORC1,which results in overall synaptogenesis and synaptic potentiation (19-21). Among other postsynaptic signaling pathways (36), the ketamine-induced synaptogenesis involves the inhibition of the eukaryotic elongation factor 2 (eEF2) kinase, leading to reduced eEF2 phosphorylation and subsequently increased BDNF translation (37). 

In anesthetic doses of ketamine, there are no increases and may be even decreases in extracellular glutamate and in glutamate cycling (38, 39). Of interest, synaptogenesis and the antidepressant effects of ketamine are also limited to subanesthetic doses (20). Hence, there is a dose-response parallel between the glutamate surge, synaptogenesis, and the antidepressant effects of ketamine. In this study, 0.75 mg/kg of ketamine was more effective than 0.5 mg/kg of ketamine may be because a greater percentage of NMDA receptors occupancy in high doses or presynaptic disinhibition of glutamatergic neurons, with increased activation of the AMPA glutamate receptor, combined with the blockade of extrasynaptic NMDA receptors; and postsynaptic activation of neuroplasticity-related signaling pathways involving BDNF and mTORC1, at higher doses. On the other hand, closer the anesthetic dose of ketamine, antidepressant effect can be reduced or even reversed, so more research is needed to find the right and more effective dose of ketamine. PET studies are recommended for this purpose. 

The latter is of particular relevance to clinical depression, which is associated with reduced prefrontal synaptic connectivity. A major form of homeostatic plasticity is “synaptic scaling,” which regulates the overall strength of neuronal synaptic connectivity. For example, a prolonged increase in neuronal activities produces a downscaling in overall synaptic strength (40). Among many other mechanisms (41), synaptic scaling is regulated by inflammatory cytokines (e.g., tumor necrosis factor) (42) and by neurotrophins [e.g., brain-derived neurotrophic factor (BDNF)], alteration in both factors has been associated with depression (43, 44). These synaptic changes are believed to result from stress-induced altered glutamate release and astroglial loss leading to neurotrophic factor deficits and sustain increase in extracellular glutamate. The excess glutamate precipitates excitotoxicity, altered synaptic strength, reduced dendritic spine density, dendritic retraction, and reduced dendritic branching in the PFC (45, 46). Identifying signaling pathways implicated in the observed stress-related synaptic dysfunction it has been found that synaptic deficits are precipitated by the reduction in neurotrophic factors such as BDNF (44) and by inhibition of the mammalian target of rapamycin complex 1 (mTORC1) signaling pathway (47). Enhancing mTORC1 signaling or increasing BDNF produces antidepressant effects (44, 47). Together these data posit that enhancement of BDNF and mTORC1 signaling lead to prefrontal synaptic formation (synaptogenesis), and reversal of stress- and depression-induced neuronal atrophy and synaptic dysconnectivity are required for efficacious antidepressant treatment. 

In this study, ketamine’s response on depression with higher intensity (Hamilton score >=30) is weaker than lower intensity that was described above is justified. A bolus injection of low-dose ketamine (0.5 mg/kg) is more effective than infusion and probably for this reason ketamine bolus method is faster than infusion crossing the blood-brain barrier and reaches a dose optimum. In high-dose ketamine (0.75 mg/kg), there was no difference between methods probably due to dose 0.75 mg/kg which is closer to optimum dose and may be masking effect of injection method.

Therefore, it seems that ketamine could theoretically play an important role in the treatment of severe and life-threatening disorders, particularly depression that requires immediate intervention, such as the use of ECT. The dose of 0.75 mg / kg dose was more effective than 0.5 mg / kg and is closer to the proper dosage and in the low dose, during bolus injection drug passes faster than infusion from the blood brain barrier and reaches an appropriate level of treatment. The present study has shown that ketamine can quickly create appropriate therapeutic effect and even to maintain its therapeutic effect for about a month but given that, the duration of ketamine gradually decline towards the end of the second month, and since depression is an episodic disease, this is another reason to start antidepressant treatment after injection of ketamine because there is a possibility of recurrence in the future. As a result, the combination therapy involves the use of ketamine due to its rapid effects associated with conventional maintenance treatments as new therapeutic protocols recommended to improve symptoms of severe depression. 

The limitations of the study are as follows: a) the small number of patients who met the requirements to participate in the study. b) Patient's unwillingness to cooperate with ketamine experimental treatment. c) Failure to timely refer patients for tests to determine the severity of depression.
